# The urinary albumin‐to‐creatinine ratio can direct personalized prevention and treatment for cardiovascular and chronic kidney disease

**DOI:** 10.1111/joim.70066

**Published:** 2026-02-03

**Authors:** Holly J. Kramer, George L. Bakris

**Affiliations:** ^1^ Departments of Public Health Sciences and Medicine, Division of Nephrology and Hypertension Loyola University Chicago Maywood Illinois USA; ^2^ Department of Medicine, Division of Nephrology University of Chicago Medicine Chicago Illinois USA

**Keywords:** albumin‐to‐creatinine ratio, cardiovascular disease, chronic kidney disease, hypertension, prevention, type 2 diabetes mellitus

## Abstract

Increased urinary albumin excretion is a strong predictor for cardiovascular events in persons with and without decreased glomerular filtration rate and can be assessed with the urinary albumin‐to‐creatinine ratio (UACR), which is a selective, sensitive, and convenient method for patients. As UACR levels ≥30 mg/g indicate heightened risk for cardiovascular disease (CVD) and chronic kidney disease (CKD) progression, this biomarker may be used to personalize preventive care. Among individuals with UACR ≥30 mg/g, reducing the UACR by at least 30% from the pretreatment (baseline) value is associated with a reduction in the risk for both cardiovascular and kidney events. Monitoring change in the UACR after drug treatment starts can be used to determine a need for medication adjustments, such as dose escalations, switching drug class, or adding further drug classes in patients with UACR ≥30 mg/g. In this review, we discuss how the biomarker UACR may be used to determine CVD and CKD risk, guide treatment, and monitor treatment response, and that the UACR is an effective tool to personalize medicine in patients with CKD.

## Graphical abstract

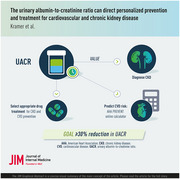

## Introduction

Cardiovascular disease (CVD) affects almost 30 million United States (US) adults and accounted for over 900,000 deaths in 2020 [1]. Interventions, such as blood pressure control, reductions in smoking prevalence, and use of cholesterol‐lowering medications, have successfully lowered CVD mortality rates over the past 60 years [2]. However, CVD burden is trending upward [3]. CVD burden and associated direct medical expenditures, which exceed $400 billion per year (2023 update), are set to increase, owing to factors such as the aging of the US population and increases in the prevalence of hypertension, obesity, and diabetes [1, 4]. Chronic kidney disease (CKD) affects more than 1 in 7 US adults, but most are unaware and are not tested [5]. Presence of CKD (particularly with significant albuminuria) increases the risk for not only kidney failure [6] but also for CVD, including myocardial infarction, heart failure, and atrial fibrillation [7]. To reduce the burden of CVD, clinicians will need to identify individuals who are at high risk for CVD and who may benefit from preventive interventions [4]. Assessment of CVD risk will need to include CKD measures, and testing should begin during early adulthood depending on comorbidities [8, 9].

In November 2023, the American Heart Association (AHA) provided a Presidential Advisory on the high prevalence of poor cardiovascular–kidney–metabolic (CKM) health among the adult population in the US and emphasized the association of poor CKM health with the high incidence of CVD and associated mortality [8]. CKM syndrome is a progressive condition due to adiposity leading to inflammation and insulin resistance [8, 10] and is interconnected with CKD. The AHA advisory included a recommendation for annual CKD testing using estimated glomerular filtration rate (eGFR) and urine albumin‐to‐creatinine ratio (UACR) starting from age 21 years for individuals with metabolic syndrome and/or subclinical or clinical CVD [8]. Because over 70% of the US adult population is now overweight or obese [11, 12], CKD testing may be operative for the majority of US adults.

Assessment of the absolute risk of CVD can help personalize the degree of prevention efforts for a given individual [13]. The recently published PREVENT equation for prediction for incident CVD and CVD subtypes differs from previous CVD risk prediction models because it includes the kidney disease measures eGFR and UACR [13]. Inclusion of the UACR significantly improves the calibration of CVD risk among individuals with increased UACR. Approximately 10% of US adults have moderate or severely increased UACR [14].

The biomarker UACR can also help personalize interventions for CVD prevention and reduce risk of CKD progression. Personalized medicine (also called precision medicine) is a medical model that aims to provide targeted prevention and treatment strategies for defined groups of individuals [15, 16]. This medical model uses an individual's current health status (“phenotype”) and genetic makeup for tailoring the right treatment strategy for the right person at the right time and/or to determine their risk for developing a disease and/or to deliver timely and targeted prevention. Biomarkers are an important aspect of personalized medicine because they can be used to predict disease prognosis, risk, and drug dose selection at the patient level. Furthermore, biomarkers can be used to identify patient populations that are more likely to benefit from interventions or experience side effects.

**Fig. 1:**
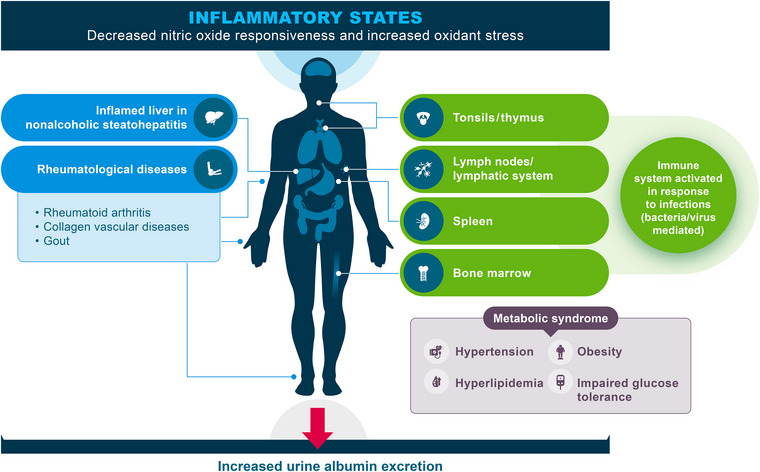
Increased urine albumin (microalbuminuria) as a marker of inflammation across multiple conditions and syndromes. Source: Adapted from Bakris and Molitch, 2014 [27].

UACR is a biomarker that can reflect early damage to the glomerular filtration barrier of the kidneys [17, 18]. The average daily urine protein excretion in adults is 80 mg/day in a healthy state (normal is considered <150 mg/day); however, albumin is approximately 15% of the total daily urine protein excretion when urine protein excretion is within the normal range [19]. Albumin is a small (66.5 kDa), negatively charged globular protein, which is normally prevented from leaking into the urine by the three layers of the glomerular capillary (endothelium, glomerular basement membrane, and podocytes) [20, 21, 22, 23, 24]. Increased albumin in the urine reflects damage to at least one of these glomerular capillary barriers, particularly the podocytes [24, 25]. More broadly, increased urine albumin excretion is an indicator of inflammation; certain diseases that have inflammation at their core are associated with increased urine albumin levels (Fig. 1) [26, 27]. Furthermore, urine albumin excretion may also fluctuate in an individual with recent exercise, acute illness, and changes in blood pressure [28, 29]. Thus, only persistently increased urine albumin excretion (≥3 months) reflects nephron damage and UACR testing should not be performed during an acute illness, such as during an active urinary tract infection [30].

### What is the UACR?

**Fig. 2:**
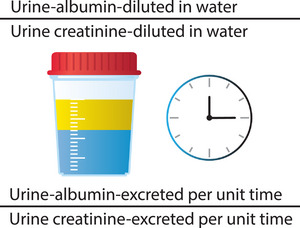
Measuring and calculating the urinary albumin‐to‐creatinine ratio.

The UACR is a ratio of urine albumin to urine creatinine in mg/g measured in a urine specimen; it reflects the amount of albumin excreted in urine in mg/g of creatinine [31]. To convert the UACR in mg/g to SI units of mg/mmol, divide the ratio by 10. The ratio is used because if it is assumed that both albumin and creatinine are diluted in the same amount of water, then any dilutional effects are cancelled out (Fig. 2). The time factor also cancels out with the use of the ratio because it is assumed that both albumin and creatinine are excreted over the same timeframe.

A UACR (after repeat testing) of <30 mg/g is considered normal to mildly increased urine albumin (previously called normoalbuminuria); a UACR of 30–299 mg/g is moderately increased urine albumin (previously called microalbuminuria); and a UACR of ≥300 mg/g is severely increased urine albumin (previously called macroalbuminuria) [18, 29]. Historically, the term microalbuminuria was used to describe moderately increased albumin excretion because urine dipsticks, which detect total urine protein excretion, do not detect these abnormalities in urine albumin excretion [30, 32].

### The UACR in personalized medicine: is the UACR the best method for measuring urine albumin?

In the following sections, we refer only to the UACR as a method for measuring urine albumin. A timed urine collection is the gold standard for assessment of urine albumin excretion, but due to patient burden and potential errors in urine collection, the UACR is the preferred method. A first morning urine sample is preferred because it shows a stronger correlation with a 24‐h urine albumin measurement compared to random collections and can minimize the effects of hydration, exercise, and posture that can all influence the UACR [33]. Both urine creatinine and albumin must be assessed because the measurement of urine albumin alone in a random urine specimen is not as sensitive or as specific as the UACR spot test. The UACR measures the amount of urine albumin excreted as a ratio relative to urine creatinine excretion, which depends on muscle mass. Among individuals with very low muscle mass, the denominator will be lower, and this may lead to an overestimation of the UACR, whereas high muscle mass can lead to an underestimation of the UACR [34, 35, 36]. In clinical scenarios of very low or high muscle mass, such as patients with severe obesity, a timed urine collection can be helpful to determine urine albumin excretion [34]. A UACR value ≥30 mg/g in a random untimed urine specimen should be confirmed in a subsequent first morning urine or random urine specimen [18].

Another method is the urine protein‐to‐creatinine ratio that can be used clinically to measure proteinuria; however, this test is less sensitive than the UACR spot test when proteinuria levels are low [18]. Mechanistically, the presence of above‐normal levels of urine albumin excretion is more likely to be associated with injuries of the glomerular filtration apparatus (when considering CKD), whereas proteinuria signifies a broader spectrum of kidney injuries, which includes tubulointerstitial injury or systemic diseases such as multiple myeloma [18]. Due to the numerous options for urine diagnostic tests, laboratories should standardize notation for UACR on order sets to limit confusion.

In this review, we discuss how the UACR is a biomarker for CKD and CVD risk and can identify treatments for slowing CKD progression and reducing CVD, regardless of the GFR.

## Methods

PubMed searches were used to find applicable articles for inclusion in all sections of this review. The following search terms were used in three separate searches: (“personalized OR personalised”) AND (“medicine OR care OR healthcare”) AND (“kidney OR renal”); (“UACR OR albumin* OR creatinine”) AND (“heart OR cardio*”); (“UACR OR albumin* OR creatinine”) AND (“renal OR kidney”) AND (“biomarker OR marker OR predict*”). Searches were limited to title only, English, humans, and past 5 years (1 January 2018–31 December 2023). These initial searches produced 375 articles. The search results were downloaded from PubMed into the EndNote 9.3.3 program, where the articles were screened using EndNote's search features as follows: removal of duplicate articles; review of article titles and removal of nonrelevant articles; review of full text and removal of nonrelevant articles. Nonrelevant articles removed included the following types of article: cancer or carcinoma; child, pediatric, and neonate; genetic bench studies; and serum albumin focus. Completed phase III, phase IV, and observational studies listed in ClinicalTrials.gov in applicable fields were also reviewed, and associated primary or secondary manuscripts were accessed and included in the reference list and cited where appropriate. Additional papers not included in the original PubMed searches were also included if recommended by the authors. Clinical treatment guideline articles applicable to CKD, diabetes, and/or hypertension were also included in the reference list.

### The UACR in personalized medicine

#### A biomarker for cardiovascular risk

Multiple studies and separate analyses have shown that an increased UACR (≥30 mg/g) is associated with heightened CVD risk, regardless of eGFR [37, 38, 39, 40]. Additionally, cardiovascular risk may be present even if the UACR is evaluated within the normal range. Results from a meta‐analysis that included over 25 million individuals from 114 global cohort studies showed that the risk of cardiovascular mortality, myocardial infarction, stroke, and heart failure increased with UACR above 10 mg/g [7]. Thus, based on this meta‐analysis, both normoalbuminuric (UACR <30 mg/g) and albuminuric (>30 mg/g) individuals may be at risk of CVD and related mortality. Although the risk of CV events associated with the UACR differed by eGFR, there was a trend toward higher levels of cardiovascular event risk with increasing UACR across all eGFR values. For example, among individuals with baseline eGFR values of 90–104 mL/min/1.73 m^2^, cardiovascular mortality increased 1.3‐fold with UACR 10–29 mg/g, 1.9‐fold with UACR 30–299 mg/g, and 2.7‐fold with UACR ≥300 mg/g relative to UACR <10 mg/g. Additionally, there was a greater associated risk of cardiovascular events among individuals with a reduced eGFR (<60 mL/min/1.73 m^2^) and higher UACR versus those without an increased UACR and reduced eGFR. For example, the risk of cardiovascular mortality was approximately 5‐fold higher in individuals with UACR values ≥300 mg/g and eGFR 15–29 mL/min/1.73 m^2^ compared with those with UACR <10 mg/g and eGFR 90–104 mL/min/1.73 m^2^. Higher UACR values also reflect a heightened risk of CKD progression to kidney failure across all eGFR levels; a similar pattern is also observed with CV risk. Thus, UACR levels reflect risk for both CV events and CKD progression.

Cohort studies and meta‐analysis highlight the importance of UACR testing and screening in patients at risk of severe CV events, which includes persons with type 2 diabetes mellitus (T2DM), older adults, those with preexisting CVD, and those with reduced eGFR. UACR can be used not only as a biomarker to predict a person's future cardiovascular risk based on their baseline/pretreatment UACR value but also as support for a more personalized approach in drug treatment selection.

#### Focus on CKD

**Fig. 3:**
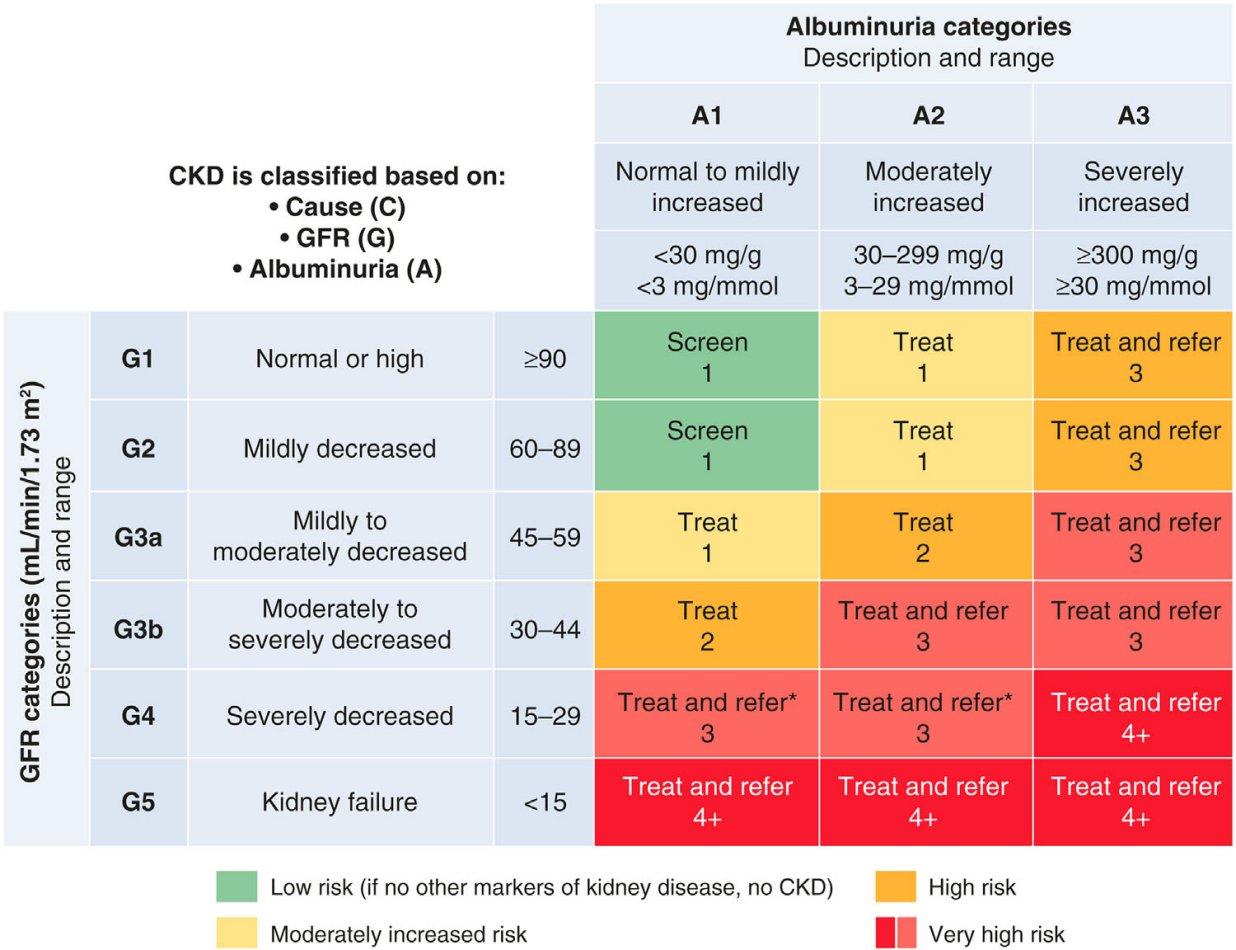
ADA‐KDIGO heatmap representing the risk for CKD progression using the GFR and albuminuria measurement categories [41]. Green: low risk (if no other markers of kidney disease, no CKD); yellow: moderately increased risk; orange: high risk; red: very high risk. The figure notes the risk of CKD progression, suggested frequency of visits, and referral to nephrology according to the eGFR and albuminuria (UACR mg/g). The numbers in the boxes are a guide to the frequency of screening or monitoring (number of times per year). Green reflects no evidence of CKD by the eGFR or albuminuria (UACR mg/g), with screening indicated once per year. For monitoring of prevalent CKD, the suggested monitoring varies from once per year (yellow) to four times or more per year (i.e., every 1–3 months [deep red]) according to risks of CKD progression and complications. The ADA‐KDIGO consensus report noted that these are general parameters only, based on expert opinion, and the patient's underlying comorbid conditions and disease state must be considered, as well as the likelihood of impacting a change in management for any individual patient [41]. ADA, American Diabetes Association; CKD, chronic kidney disease; eGFR, estimated glomerular filtration rate; GFR, glomerular filtration rate; KDIGO, kidney disease: improving global outcomes; UACR, urinary albumin‐to‐creatinine ratio.

CKD is diagnosed by the presence of persistently elevated UACR and/or decreased eGFR (<60 mL/min/1.73 m^2^), or other manifestations of kidney damage for ≥3 months [18]. Both eGFR and UACR are necessary for accurate CKD staging, estimation of the risk of progression, and guidance on frequency of visits and referral to nephrology services (Fig. 3) [41]. Approximately 40% of patients with diabetes will develop CKD in their lifetime [42], and the American Diabetes Association/Kidney Disease: Improving Global Outcomes (ADA/KDIGO) 2022 consensus report recommends that clinicians monitor both eGFR and UACR at least annually in patients with T2DM, typically using the UACR spot test [41]. For those with type 1 diabetes, annual testing for CKD should commence after a diabetes duration of ≥5 years [41]. For people with established CKD, UACR and eGFR should be monitored 1–4 times per year depending on the stage of disease [29]. CKD can occur in the absence of increased UACR (or albuminuria) [43, 44] and occurs in approximately one‐third of adults with diabetes [45]. Indeed, a phenotype called the “non‐proteinuric/non‐albuminuric diabetic kidney disease” phenotype has been used to describe this clinicopathologic phenomenon [46]. Nephron loss in the absence of increased UACR may be the result of arteriosclerosis, global glomerular scarring, and interstitial fibrosis [47], which is likely to be influenced by comorbidities and medication use. Absence of increased UACR in people with CKD and diabetes may occur (regression of albuminuria phenotype), which may portend to a slower rate of CKD progression [46], although not always [48]. Detection of moderately to severely increased UACR via routine testing facilitates the implementation of medical interventions that can prevent or slow the progression of CKD and prevent kidney failure [49, 50, 51]. Pharmacologic CKD‐focused management is not included in guideline recommendations for patients with diabetes who have a UACR <30 mg/g and a normal eGFR, as this does not indicate CKD [41].

Chronic hypertension can also lead to CKD, but treatment guidelines in this field do not consistently recommend both CKD testing for eGFR and UACR [52, 53, 54]. The 2017 American College of Cardiology (ACC)/AHA guideline recommends a urinalysis and assessment of eGFR as part of routine testing to evaluate primary hypertension, whereas UACR testing is considered optional, even if hypertension is deemed resistant and/or due to secondary causes [52]. However, in patients with known CKD and a UACR ≥300 mg/g, renin–angiotensin–aldosterone system (RAAS) blockers (angiotensin‐converting enzyme inhibitors [ACEIs] or angiotensin II receptor blockers [ARBs]) are recommended as first‐line agents for hypertension. Thus, UACR testing in adults with hypertension may help guide the selection of blood pressure–lowering medications. Incidence of moderately to severely increased albuminuria, or a UACR ≥30 mg/g, may be similar in individuals with hypertension and no diabetes (5‐year incidence, 22%) compared with those with diabetes (24%), yet UACR testing remains very low in adults with hypertension [55].

### Assessment of cardiovascular, kidney, and metabolic (CKM) health

**Fig. 4:**
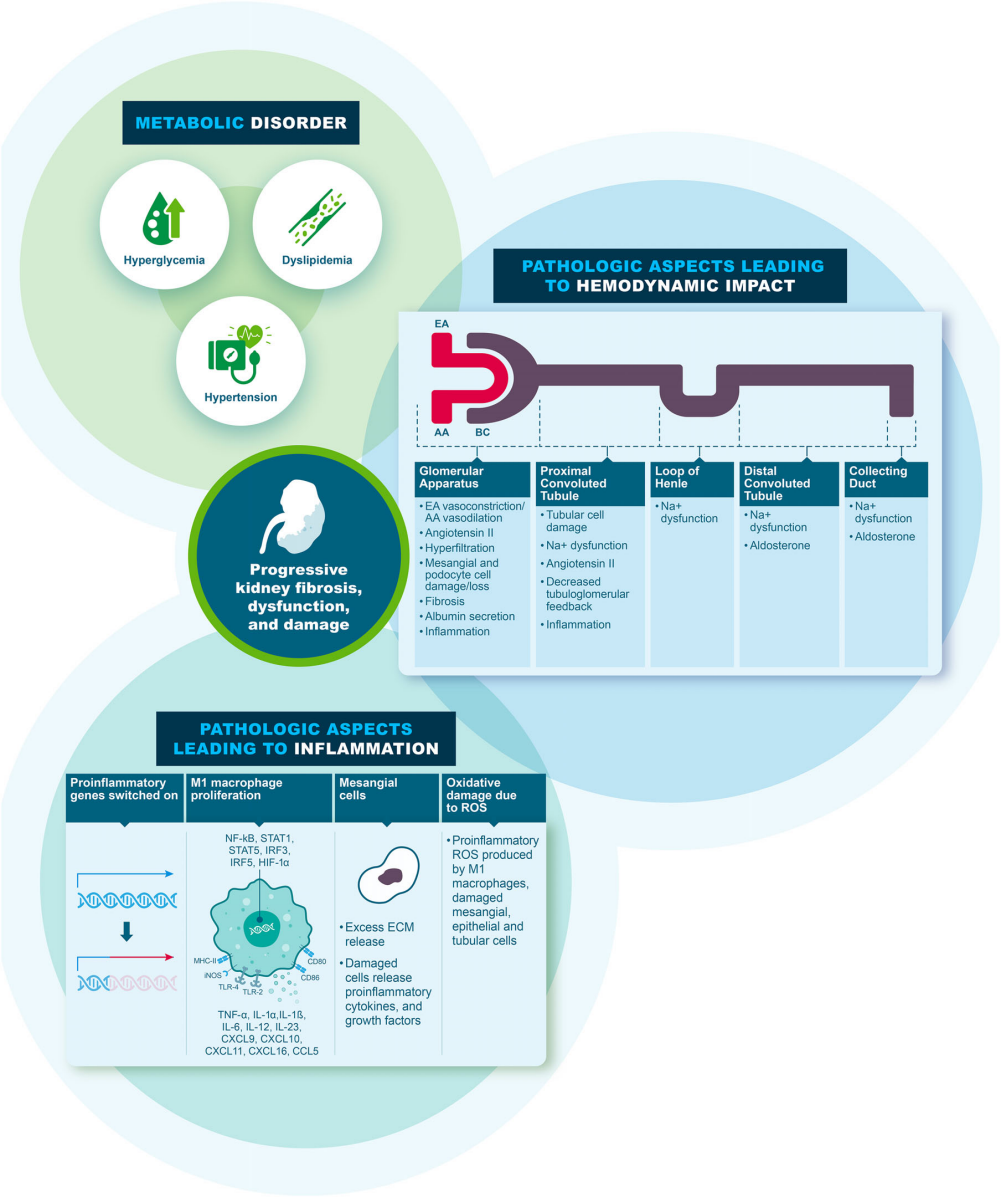
Pathophysiologic changes caused by prolonged hyperglycemia and hypertension leading to chronic kidney disease [24, 57, 58, 59, 60, 61, 62, 63, 64, 65, 66, 67, 68, 69, 70, 71, 72, 73, 74, 75, 76, 77]. AA, afferent arteriole; BC, Bowman's capsule; CCL, chemokine (C–C motif) ligand; CD, cluster of differentiation; CXCL, chemokine (C–X–C motif) ligand; EA, efferent arteriole; ECM, extracellular matrix; HIF, hypoxia‐inducible factor; IL, interleukin; iNOS, inducible nitric oxide synthase; IRF, interferon regulatory factor; MHC, major histocompatibility complex; Na+, sodium; NF‐κB, nuclear factor kappa beta; ROS, reactive oxygen species; STAT, signal transducer and activator of transcription; TLR, toll‐like receptor; TNF, tumor necrosis factor.

A CKD‐prevention priority for people with CKM syndrome is to maintain normal levels of blood glucose and normal blood pressure given that high blood glucose and high blood pressure [56] can lead to detrimental effects on glomerular capillaries and kidney function (Fig. 4) [24, 57, 58, 59, 60, 61, 62, 63, 64, 65, 66, 67, 68, 69, 70, 71, 72, 73, 74, 75, 76, 77]. Albuminuria in the setting of metabolic syndrome reflects multiple pathologic processes, which may include increased intraglomerular capillary pressure, decreased podocyte density, glucose‐mediated toxicity, inflammation, and overactivation of mineralocorticoid receptors [78, 79, 80, 81, 82, 83]. These processes that can lead to increased urine albumin excretion can be addressed in part via glucose and blood pressure control, which can be achieved through lifestyle changes such as diet, exercise, smoking cessation, lifestyle optimization, and maintaining a healthy weight, and, when necessary, medications [29, 56]. Weight loss itself leads to reductions in urine albumin excretion. In fact, weight loss via lifestyle modifications, bariatric surgery, or medication lowers UACR levels in various populations, including those with T2DM, hypertension, and/or CKD [84, 85, 86].

Based on information in treatment guidelines and clinical study analyses in T2DM and/or hypertension, it is clear that the UACR not only has an important screening and diagnostic role in CKD but can also be modified by lifestyle changes and pharmacologic management as outlined hereafter.

### UACR in personalized medicine: a biomarker for treatment decisions in CKD

The ADA's standards of care in diabetes and CKD state that a UACR reduction of ≥30% is recommended to slow CKD progression for individuals with severely increased urine albumin (UACR ≥300 mg/g) [29]; however, some studies have shown a clinically significant benefit with a ≥30% reduction even when the pretreatment UACR is <300 mg/g. Indeed, the concentration of urinary albumin (via the UACR, typically alongside the eGFR) is used as a marker for when add‐on therapies should be used. RAAS blockers reduce not only systemic blood pressure but also intraglomerular capillary pressure and, consequently, are considered first‐line therapy for blood pressure treatment in individuals with moderately to severely increased UACR regardless of diabetes status [29, 41, 52, 87, 88]. Moreover, an RAAS blocker is recommended in CKD where UACR is moderately or severely increased with or without diabetes and/or hypertension being present [18]. Moreover, for those with diabetes and hypertension, RAAS blockers are also considered first‐line therapies for lowering blood pressure and to prevent the progression of kidney disease and reduce cardiovascular events when the UACR is ≥30 mg/g [29].

### UACR as a marker of treatment response to SGLT2 inhibitor and/or ns‐MRA therapy in patients with CKD

In addition to having an important CKD diagnostic and screening role, the UACR can be used as a marker of drug treatment response. We have already noted that a UACR reduction of at least 30% from the pretreatment baseline suggests a treatment benefit. Often such a reduction in UACR translates into cardiovascular and kidney benefits. Not all patients with CKD have increased UACR, but a higher UACR value indicates increased risk for CKD progression. If the UACR does not decrease by ≥30% of the baseline value with use of RAAS blockade and blood pressure control and/or remains persistently increased, then we recommend other medication classes be added to ACEIs or ARBs. Assuming blood pressure is controlled to levels <130/80 mmHg, additional medications that could be added include a sodium‐glucose cotransporter‐2 (SGLT2) inhibitor (if not already added), the nonsteroidal mineralocorticoid receptor antagonist (ns‐MRA) finerenone in the setting of T2DM, and/or a glucagon‐like peptide‐1 receptor agonist (GLP‐1 RA) in the setting of obesity and/or T2DM [18, 29, 56] (Fig. S1). In addition to RAAS blockade, SGLT2 inhibitors, ns‐MRA, and GLP‐1 RA may be used in combination to lower UACR and reduce risk of CKD progression and negative CV outcomes, including heart failure [89]. Lifestyle interventions should also be utilized including medical nutrition therapy.

SGLT2 inhibitors reduce the UACR and have shown cardiovascular‐ and kidney‐protective effects to varying degrees in clinical trials involving patients with CKD only or with CKD and diabetes [90, 91, 92, 93, 94]. Additionally, these effects were observed across UACR subgroups, with some analyses showing bigger cardiovascular‐ and kidney‐protective effects at higher baseline UACRs. The EMPA‐KIDNEY study assessed the effect of the SGLT2 inhibitor empagliflozin in patients with an eGFR 45 to <90 mL/min/1.73 m^2^ with a UACR ≥200 mg/g, or with an eGFR 20 to <45 mL/min/1.73 m^2^ with any level of UACR [94]. Overall, empagliflozin reduced the relative risk of the primary composite outcome (progression of kidney disease or death from cardiovascular causes) by 28% versus placebo. At the end of the treatment period, the UACR was 19% lower in the empagliflozin group than in the placebo group. However, the protective effects of empagliflozin were not equally spread across all baseline UACR subgroups (Table 1) [94]. In a post hoc analysis from the Canagliflozin and Renal Events in Diabetes with Established Nephropathy Clinical Evaluation (CREDENCE) trial, the SGLT2 inhibitor canagliflozin reduced the UACR across all UACR categories in patients with T2DM and CKD, but there were greater proportional reductions in the higher UACR categories versus placebo (Table 1) [90]. In another post hoc analysis of these data, early, sustained reductions in the UACR with canagliflozin (vs. placebo) were independently associated with beneficial long‐term kidney and cardiovascular outcomes (vs. placebo) (Table 1) [91]. In an analysis of data from the DAPA‐DKD trial, the SGLT2 inhibitor dapagliflozin reduced the relative risks for kidney and cardiovascular composite endpoints in patients with CKD (with or without T2DM) across all subgroups of UACR (≤1000, >1000 to ≤3500, and >3500 mg/g) and eGFR (<30, ≥30 to <45, and ≥45 mL/min/1.73 m^2^) (all *p* for interaction >0.10); however, the absolute benefit for the kidney‐specific secondary endpoints was greater in subgroups with a high UACR at baseline [93]. Thus, based on these analyses, individuals with a higher pretreatment baseline UACR level (typically >300 mg/g) may gain the most benefit with an SGLT2 inhibitor.

**Table 1:** Summary of published efficacy data from outcomes trials in patients with CKD or CV disease, which investigated the impact on UACR levels.

Trial ID/study name	Population	Treatment groups	Primary outcome measure/primary objective of the post hoc/secondary analysis	UACR endpoint results
RCTs/analyses in CKD				
EMPA‐KIDNEY [95]	CKD at risk of progression (with or without diabetes)	Empagliflozin + ACEI/ARB versus placebo + ACEI/ARB	Primary outcome measure: progression of kidney disease or death from CV causes (primary composite endpoint)	UACR reduction 19% lower with empagliflozin versus placebo (95% CI: 15–23) Primary outcome by UACR (mg/g) (prespecified) subgroup: <30: HR 1.01 (95% CI: 0.66–1.55) ≥30 to ≤300: HR 0.91 (95% CI: 0.65–1.26) >300: HR 0.67 (95% CI: 0.58–0.78)
CREDENCE secondary and post hoc analyses [91, 92]	T2DM and CKD at high risk of progression	Canagliflozin + ACEI/ARB versus placebo + ACEI/ARB	Primary objective of the secondary analysis (Jardine et al. [91])—the association between baseline UACR and the effects canagliflozin on efficacy and safety outcomes in CREDENCE Primary objective of the post hoc analysis (Oshima et al. [92])—canagliflozin's effect on albuminuria and how early change in albuminuria is associated with the primary kidney outcome, major adverse CV events, and HHF, or CV death	UACR reductionCanagliflozin versus placebo: ↓31% (95% CI: 26–35) OR for >30% reduction in UACR at week 26 2.69 (95% CI: 2.35–3.07) By UACR subgroups: ≤1000 mg/g: ↓35% (95% CI: 29–39) >1000 to <3000 mg/g: ↓29% (95% CI: 21–35) ≥3000 mg/g: ↓14% (95% CI: −2 to 28)
DAPA‐DKD post hoc analysis [94]; prespecified analysis [93]	CKD with and without T2DM	Dapagliflozin + ACEI/ARB versus placebo + ACEI/ARB	Primary objective of the post hoc analysis (Waijer et al. [94]) to assess the efficacy and safety of dapagliflozin according to baseline KDIGO risk categories Primary objective of the prespecified analysis (Jongs et al. [93]) to assess the effects of dapagliflozin on albuminuria in patients with CKD with/without T2DM DAPA‐CKD trial	UACR reduction (overall) ↓29.3% (vs. placebo) (95% CI: −33.1 to −25.2); *p* < 0.0001 UACR reduction (with or without diabetes) −35.1 (relative to placebo) with diabetes; −14.8% (relative to placebo) without diabetes Primary outcome by UACR (mg/g) (prespecified) subgroupUACR subgroups (diabetes): ≤1000 mg/g: HR 0.59 (95% CI: 0.38–0.92) >1000 to ≤3500 mg/g: HR 0.58 (95% CI: 0.43–0.78) >3500 mg/g: HR 0.66 (95% CI: 0.45–0.97) *p* for interaction = 0.90 UACR subgroups (without diabetes): ≤1000 mg/g: HR 0.42 (95% CI: 0.22–0.83) >1000 to ≤3500 mg/g: HR 0.60 (95% CI: 0.36–0.97) >3500 mg/g: HR 0.34 (95% CI: 0.12–0.92) *p* for interaction = 0.42
FIDELIO‐DKD [96]	T2DM and CKD treated with an ACEI/ARB (max dose without unacceptable side effects)	Finerenone + ACEI/ARB versus placebo + ACEI/ARB	Primary composite outcome: time to kidney failure, a sustained decrease of ≥40% in eGFR from baseline over a period of ≥4 weeks, or death from renal causes (finerenone vs. placebo)	UACR reduction at 4 monthsFinerenone versus placebo: ↓31% ratio of least‐squares mean change from baseline, 0.69 (95% CI: 0.66–0.71)
FIGARO‐DKD [97]	T2DM and CKD treated with an ACEI/ARB (max dose without unacceptable side effects)	Finerenone + ACEI/ARB versus placebo + ACEI/ARB	Primary composite outcome: time to death from CV causes, nonfatal MI, nonfatal stroke, or H HF (finerenone vs. placebo)	UACR reduction at 4 monthsFinerenone versus placebo: ↓32% ratio of least‐squares mean change from baseline, 0.68 (95% CI: 0.65–0.70)
FIDELITY pooled analysis [98]	T2DM and CKD treated with an ACEI/ARB (max dose without unacceptable side effects)	Finerenone + ACEI/ARB versus placebo + ACEI/ARB	Outcome measures for this pooled analysis: composite CV outcome: time to CV death, nonfatal MI, nonfatal stroke, or HHF; and composite kidney outcome time to first onset of kidney failure, sustained ≥57% decrease in eGFR	UACR reduction at 4 monthsFinerenone versus placebo: ↓32% ratio of least‐squares mean change from baseline, 0.68 (95% CI: 0.66–0.70)
FIDELITY‐HF subgroup analysis [99]	T2DM and CKD treated with an ACEI/ARB (max dose without unacceptable side effects)	Finerenone + ACEI/ARB versus placebo + ACEI/ARB	Primary objective of the subgroup analysis: to evaluate the effects of finerenone on HF outcomes by eGFR and/or UACR subgroups	Reduced risk of first HHF by baseline UACR subgroups (finerenone vs. placebo) By 29% UACR <300 mg/g (HR 0.71 [95% CI: 0.52–0.97]) By 17% UACR ≥300 mg/g (HR 0.83 [95% CI: 0.68–1.00]) *p* for interaction, 0.36 Similar results for composite of CV death or first HHF, recurrent HHF, and the composite of CV death or recurrent HHF with lower event rates with a UACR <300 versus ≥300 mg/g and with a trend toward larger risk reductions with finerenone versus placebo in patients in lower versus higher baseline UACR subgroups but no evidence of heterogeneity (*p* for interactions, >0.1)
FIDELTY‐kidney subgroup analysis [100]	T2DM and CKD treated with an ACEI/ARB (max dose without unacceptable side effects)	Finerenone + ACEI/ARB versus placebo + ACEI/ARB	Primary objective of the subgroup analysis: to evaluate the kidney benefits and kidney safety of finerenone in patients with CKD stage 1–4 with moderately to severely elevated albuminuria and T2DM	Composite kidney outcome by baseline UACR. Larger effect with UACR ≥300 but interaction showed no evidence of heterogeneity 30 to <300 mg/g: HR 0.94 (95% CI: 0.60‒1.47) ≥300 mg/g: HR 0.75 (95% CI: 0.65‒0.87) *p* for interaction, 0.6673
FLOW trial [105]	T2DM and CKD	Semaglutide versus placebo (randomization stratified by SGLT2 inhibitor use at baseline)	Primary outcome: composite of kidney events that included kidney failure, eGFR <15 mL/min/1.73 m^2^, a ≥50% reduction in eGFR from baseline, or death from kidney or CVD	UACR reduced by 40% with semaglutide versus 12% with placebo (3.4 years) (HR 0.68 [95% CI: 0.62–0.75])
RCTs/analyses in CVD				
EMPEROR‐Pooled secondary analysis [101]	Chronic HF NYHA class II–IV	Empagliflozin versus placebo	Primary objective of the pooled secondary analysis: to analyze the association of empagliflozin with study outcomes across baseline levels of albuminuria and change in albuminuria in patients with HF across a wide range of ejection fraction levels	Risk of UACR category shift (empagliflozin versus placebo) ≤300 upshift to >300 mg/g: ↓19% (HR 0.81 [95% CI: 0.70–0.94]; *p* = 0.005) >300 downshift to ≤300 mg/g: ↑31% (HR 1.31 [95% CI: 1.07–1.59]; *p* = 0.009)
EMPA‐REG OUTCOME post hoc analysis [102]	T2DM and established CVD	Empagliflozin + SOC versus placebo + SOC	Primary objective of the post hoc analysis: to evaluate whether an early change in albuminuria on treatment with empagliflozin is associated with long‐term CV and renal outcomes and whether this association is independent of the change in eGFR and other CV risk factors	Association between baseline UACR and CV and renal outcomes (all patients from both treatment arms) Compared with the low UACR group (<30 mg/g), the intermediate‐high (>300–1000 mg/g) and high (>1000 mg/g) UACR groups experienced significantly more MACE, CV death/HHF, and renal outcomes UACR reduction from BL to week 12Empagliflozin (vs. placebo): ↓18% (95% CI: 14–22) OR for >30% reduction in UACR at week 12 1.42 (95% CI: 1.27–1.58) By UACR subgroups ≥30 mg/g: ↓34% (95% CI: 26–41)
TOPCAT secondary analysis [103]	HFpEF	Spironolactone versus placebo	Primary objective of the analysis: to examine the association of UACR with the primary outcome from TOPCAT (CV death, aborted cardiac arrest, or HHF) and its individual components, all‐cause mortality, and several safety endpoints	UACR reduction at 1 yearSpironolactone versus placebo overall (vs. placebo) ↓39% GMR: 0.61 (95% CI: 0.49–0.77); *p* < 0.001 Rate (per 100 PY) of CV mortality, aborted cardiac arrest, or HHF by UACR subgroups (all patients): ≥30 versus <30 mg/g: 14.3 versus 8.4 (HR 1.47 [95% CI: 1.15–1.86]) ≥300 versus <30 mg/g: 16.8 versus 8.4 (HR 1.67 [95% CI: 1.22–2.28]) GMR (95% CI) by UACR subgroups: <30 mg/g: 0.65 (95% CI: 0.48–0.89); *p* = 0.006 ≥30 mg/g: 0.78 (95% CI: 0.55–1.11); *p* = 0.17 ≥300 mg/g: 0.24 (95% CI: 0.10–0.56); *p* = 0.001
MIRAD [104]	T2DM at high risk of or with established CVD	Eplerenone + antihypertensives versus dose‐matched placebo + antihypertensives	Primary objective of the analysis: to examine the change in UACR and 24‐h ambulatory blood pressure with eplerenone versus placebo using data from the MIRAD trial (prespecified secondary endpoints)	UACR reduction at 26 weeksEplerenone versus placebo ↓34% (95% CI: −51 to −12); *p* = 0.005
CREDENCE [141]	T2DM and high‐risk CV risk	Canagliflozin versus placebo	Effects of canagliflozin on CV outcomes according to baseline eGFR and UACR	Rates of CV death or HHF increased as eGFR declined and/or UACR increased Consistent results across eGFR and UACR categories (all *p* interaction >0.40)
DECLARE‐TMI [139]	T2DM and CKD	Dapagliflozin versus placebo	Dual primary end points: major adverse CV events (myocardial infarction, stroke, and CV death) and the composite of CV death or HHF	Effect of dapagliflozin (vs. placebo) on the relative risk of a composite of CV death and HHF and of major adverse cardiovascular events was similar However, effect of dapagliflozin on the relative risk for CV events was consistent across UACR groups (<30 vs. ≥30 mg/g)
VERTIS CV [142]	T2DM and atherosclerotic CV disease	Ertugliflozin versus placebo	Included time to first event of HHF	HF hospitalization ∼50% lower in both moderately increased UACR (HR 0.51; 95% CI 0.34, 0.77) and severely increased UACR (HR 0.58; 95% CI 0.35, 0.95) No significant association for HF hospitalization among participants with UACR <30 mg/g (HR 1.12; 95% CI 0.69, 1.83)

*Note*: Age, previous history of CVD, systolic blood pressure, UACR, hemoglobin, body weight, albumin, eGFR, and randomized treatment assignment.

Abbreviations: ACEI, angiotensin‐converting enzyme inhibitor; ARB, angiotensin II receptor blocker; CI, confidence interval; CKD, chronic kidney disease; CREDENCE, Canagliflozin and Renal Events in Diabetes with Established Nephropathy Clinical Evaluation; CV, cardiovascular; CVD, cardiovascular disease; eGFR, estimated glomerular filtration rate; ESKD, end‐stage kidney disease; GMR, geometric mean ratio; HF, heart failure; HFpEF, heart failure with preserved ejection fraction; HHF, hospitalization for heart failure; HR, hazard ratio; MI, myocardial infarction; PY, patient‐years; RCT, randomized, controlled trial; SOC, standard of care; T2DM, type 2 diabetes mellitus; UACR, urinary albumin‐to‐creatinine ratio.

Similarly, clinical trials of the ns‐MRA finerenone involved adult patients with T2DM and UACR ≥30 mg/g [95, 96]. The FIDELIO‐DKD and FIGARO‐DKD placebo‐controlled clinical trials (and the prespecified FIDELITY pooled analysis [based on FIDELIO and FIGARO]) both showed cardiovascular and kidney benefit of finerenone (vs. placebo), with UACR reductions of ∼30% (Table 1). In the FIGARO‐DKD study, the UACR was reduced by 32% after 4 months in participants randomized to finerenone [97], and the UACR reduction was linked with cardiovascular risk reduction. In an analysis of the FIDELITY data, finerenone treatment benefits in the composite cardiovascular outcomes were noted in the UACR 30 to <300 and ≥30 mg/g subgroups, although larger treatment effects were present in the higher baseline UACR subgroup (≥300 mg/g) (*p* interaction 0.41) [98]. There was no difference across the UACR and eGFR subgroups in terms of the effect of finerenone on heart failure–related outcomes (*p* interaction >0.10), although there was a trend toward lower risk of heart failure–related outcomes with finerenone (vs. placebo) in those with a UACR <300 mg/g and those with an eGFR ≥60 mL/min/1.73 m^2^ [99]. For the kidney outcomes in FIDELITY, finerenone reduced the risk of the composite kidney outcome across UACR subgroups (30 to <300 and ≥300 mg/day) but with a degree of uncertainty in the hazard reduction due to a smaller absolute risk in the 30 to <300 mg/g UACR subgroup (*p* interaction 0.6673) [100]. To further extend these findings, results from a mediation analysis of the FIDELITY database, in keeping with the ADA 2024 guidelines, showed a sustained ≥30% reduction in UACR at 4 months, corresponding to a significant slowing of CKD progression and reduced risk of cardiovascular events [101]. Such data provide important evidence supporting UACR reduction as a biomarker predicting outcomes in diabetic kidney disease with increased UACR.

Emerging data show that some GLP‐1 RAs can lower the risk for both CVD and CKD progression in patients with CKD and obesity and/or T2DM. This drug class of incretin mimetics includes exenatide, liraglutide, dulaglutide, semaglutide, and tirzepatide, a dual GLP‐1 RA‐glucose‐dependent insulinotropic polypeptide agonist [102, 103, 104]. For example, semaglutide and high‐dose liraglutide are approved by the US Food and Drug Administration (FDA) for the treatment of obesity, or excess weight in the setting of metabolic syndrome [105, 106, 107]. The 2024 ADA guidelines recommend use of a GLP‐1 RA for cardiovascular risk reduction in adults with T2DM with, or at risk for, atherosclerotic CVD (ASCVD) or kidney disease [108]. In addition to promoting weight loss, GLP‐1 RAs lower CVD risk via multiple effects including increasing natriuresis, improved glucose control and lipid metabolism, and reduced platelet activation, oxidative stress, and inflammation [109, 110, 111, 112, 113]. Weight loss with this drug class occurs due to delayed gastric emptying and includes activation of arcuate pro‐opiomelanocortin neurons in the hypothalamus leading to reduced appetite [114, 115, 116, 117].

Use of GLP‐1 RAs reduces the UACR and improves kidney outcomes in adults with T2DM and CKD, as demonstrated in the placebo‐controlled FLOW trial with the GLP‐1 RA semaglutide [118]. The primary outcome of the FLOW trial was a composite of kidney events that included kidney failure, eGFR <15 mL/min/1.73 m^2^, a ≥50% reduction in eGFR from baseline, or death from kidney or CVD. A total of 3533 adults with T2DM and CKD, defined by (1) an eGFR of 50 to 75 mL/min/1.73 m^2^ and a UACR ratio >300 to <5000 mg/g or (2) an eGFR of 25 to <50 mL/min/1.73 m^2^ and a UACR of >100 to <5000 mg/g, were randomly assigned to receive either semaglutide 1 mg weekly (target maintenance dose) or placebo. Approximately 68% of participants had a UACR of ≥300 mg/g, and the mean eGFR was 47 mL/min/1.73 m^2^. After a median follow‐up of 3.4 years, the UACR declined by 40% with semaglutide compared with 12% with placebo (Table 1), and the risk of the composite outcome was 24% lower with semaglutide versus placebo (hazard ratio [HR] 0.76 [95% confidence interval (CI): 0.66–0.88]; *p* = 0.0003). The relative risk of cardiovascular events, defined as death from CVD, nonfatal myocardial infarction, stroke, or death from any cause, was 18% lower with semaglutide than with placebo (HR 0.82 [95% CI: 0.68–0.98]; *p* = 0.029) [118]. These cardiovascular outcome effects with semaglutide are similar to those observed with liraglutide in the placebo‐controlled Liraglutide Effect and Action in Diabetes: Evaluation of cardiovascular outcome Results (LEADER) trial, which included 9340 randomized adults with T2DM and high CVD risk [119]. After a median follow up of 3.8 years, the primary composite cardiovascular outcome, defined as death from cardiovascular causes, nonfatal myocardial infarction, or nonfatal stroke, compared with placebo, was 13% lower in the liraglutide group (HR 0.87 [95% CI: 0.78–0.97]; *p* < 0.001 [noninferiority] or *p* = 0.01 [superiority]). The benefits of the GLP‐1 RA drug class on CVD events are likely mediated via its effects on weight loss, improving glucose control, and lowering blood pressure. However, results from a mediation analysis of the LEADER trial suggest that lowering of UACR with liraglutide may account for around 30% of its benefits on CVD risk reduction [120].

These studies in CKD (and in T2DM with high cardiovascular risk) demonstrate the usefulness and prognostic importance of the medications that reduce the UACR, including SGLT2 inhibitors, ns‐MRAs, and GLP‐1 RAs (based on the UACR value before treatment). The UACR should be assessed before and after initiation of one of these medication classes to measure response to treatment. Furthermore, early clinically relevant UACR reductions are an important element of the beneficial effect of these drug classes (e.g., canagliflozin for SGLT2 inhibitors) [91, 101]. Thus, using the UACR as a predictor of treatment response in CKD could prove invaluable in deciding which drug treatment might work best for a patient, as well as in determining the need to adjust therapy to provide greater protection against kidney and cardiovascular risk as part of a personalized treatment approach.

### UACR to identify heart failure phenotypes and guide treatment

It is established that even a low UACR level is a marker of inflammation, and recently it has also been found to be a risk marker for heart failure [27, 121]. Kidney disease commonly occurs in patients with heart failure and is strongly associated with reduced survival in this high‐risk population [122]. Conversely, heart failure is a cardiac problem often seen in kidney disease [123]. Hypertension, obesity, diabetes, and physical inactivity are common and often coexist among individuals with CKD and/or heart failure with preserved ejection fraction (HFpEF) [124, 125, 126]. Additionally, reduced eGFR impairs salt handling and increases blood pressure, which can lead to HFpEF [125, 126]. Furthermore, CKD leads to afterload‐mediated myocardial dysfunction because of vasculature stiffness [124, 125]. HFpEF is a common cause for exercise intolerance among older adults and is linked with obesity but remains underdiagnosed [124].

Recommended treatment for HFpEF includes therapies such as an SGLT2 inhibitor and/or a steroidal MRA, and more recently, the ns‐MRA finerenone [127, 128]. The 2022 AHA/ACC/HFSA Guideline for the Management of Heart failure now includes SGLT2 inhibitors as one of four medication classes recommended for heart failure with reduced (dapagliflozin [129] and empagliflozin [130]) or mildly reduced ejection fraction and for HFpEF (empagliflozin) [127]. Therapy may also be tailored to specific HFpEF phenotypes that can be determined by identifying the presence of CKD, which requires, by definition, assessment of UACR levels. The lung congestion/metabolic risk phenotype of HFpEF is the most prevalent phenotype and is associated with obesity and CKD [131, 132, 133]. These patients often need diuresis as well as treatment for their CKD such as an SGLT2 inhibitor (if not already taken for heart failure) and/or an ns‐MRA (currently only finerenone) as appropriate [29, 127]. If HFpEF diagnosis is confirmed, an SGLT2 inhibitor should be started unless this drug class is contraindicated [124, 127]. Thus, identifying CKD with both eGFR and UACR testing should be part of the routine management of people with HFpEF. Use of a GLP‐1 RA with proven CKD and CVD beneficial effects, such as semaglutide, may reduce symptoms and improve physical functioning in patients with obesity and HFpEF [118, 134, 135].

### Lowering the UACR reduces rate of cardiovascular events

#### Clinical experience with SGLT2 inhibitors

Results from a post hoc analysis of the EMPEROR‐Pooled analysis in heart failure patients (data from EMPEROR‐Reduced and EMPEROR‐Preserved clinical trials) found that empagliflozin decreased the likelihood of the UACR increasing to a higher UACR category and increased the likelihood of albuminuria regression versus placebo [136]. Furthermore, empagliflozin was associated with a reduction in the primary composite endpoint of cardiovascular death or first heart failure hospitalization irrespective of UACR levels at baseline. Lowering of the UACR is also associated with reduction in cardiovascular mortality (Table 1).

Empagliflozin has also demonstrated benefits of early UACR reduction (within 12 weeks) in a post hoc analysis of the EMPA‐REG OUTCOME clinical trial. In this analysis, short‐term reductions in the UACR were statistically significantly associated with a decreased risk of long‐term cardiovascular and kidney outcomes in people with diabetes and established CVD treated with empagliflozin [137]. Treatment with empagliflozin reduced the UACR from baseline to week 12 by 18% and increased the likelihood of achieving a >30% UACR reduction (all UACR categories) versus placebo (*p* < 0.001). During 3 years of follow‐up, each 30% decrease in the UACR during the first 12 weeks of empagliflozin treatment was significantly associated with a lower risk of major cardiovascular events, cardiovascular deaths, or hospitalizations for heart failure.

In a post hoc analysis of the DECLARE‐TIMI58 trial, the effect of dapagliflozin on the relative risk for cardiovascular events was similar across the eGFR (<60 and ≥60 mL/min/1.73 m^2^) and UACR (<30 and ≥30 mg/g) subgroups in patients with T2DM with/at risk for ASCVD; the greatest absolute benefit for the composite of cardiovascular death or hospitalization for heart failure was in patients who had both reduced eGFR and UACR with dapagliflozin treatment [138]. Where assessed, these studies with empagliflozin and dapagliflozin SGLT2 inhibitors may reduce the UACR between 19% and 35%—a clinically relevant reduction in some cases (based on ADA 2024 guidelines)—across UACR categories and early after treatment start for some patients (Table 1).

The CANagliflozin cardiovascular Assessment Study (CANVAS) Program pooled data from two clinical trials that evaluated the effects of canagliflozin on the composite outcome of CV death, nonfatal myocardial infarction, and nonfatal stroke in adults with T2DM and high CVD risk [139]. Overall, canagliflozin was associated with a significantly lower risk of the composite outcome (HR 0.86 [95% CI: 0.75–0.97]). Canagliflozin use was also associated with lower risk of UACR progression (HR 0.73 [95% CI: 0.67–0.79]) [139]. An additional analysis pooled data from the CANVAS Program and the CREDENCE trial to assess the effects of canagliflozin on CV outcomes by CKD status [140]. Use of canagliflozin was associated with significantly lower risk of both heart failure hospitalization and CV death regardless of baseline UACR or eGFR and no significant differences were noted across subgroups (Table 1).

The Evaluation of Ertugliflozin Efficacy and Safety Cardiovascular Outcomes Trial randomized 8246 adults with T2DM and CVD to the SGLT2 inhibitor ertugliflozin (5, 15 mg) or placebo and followed them for heart failure hospitalization and a composite outcome of heart failure hospitalization or CV death [141]. In this trial population, approximately 24% had heart failure. Participants who received ertugliflozin showed significantly lower risk of heart failure hospitalization (HR 0.70 [95% CI: 0.54–0.90]) but findings differed by UACR subgroups (*p* = 0.04 for interaction). Compared to placebo, heart failure hospitalization was approximately 50% lower among trial participants with both moderately increased UACR (HR 0.51 [95% CI: 0.34–0.77]) and severely increased UACR (HR 0.58 [95% CI: 0.35–0.95]) randomized to ertugliflozin (Table 1). In contrast, no significant association was noted with ertugliflozin allocation and heart failure hospitalization among participants with UACR <30 mg/g (HR 1.12 [95% CI: 0.69–1.83]). No differences in major adverse cardiovascular events defined as cardiovascular death or nonfatal myocardial infarction [141].

#### Clinical experience with the combination SGLT1/2 inhibitor sotagliflozin

Similarly, a double‐blinded trial examined the effectiveness of the SGLT2 inhibitor sotagliflozin for lowering risk of a composite cardiovascular endpoint of CVD death, heart failure hospitalization, and urgent visits for heart failure [142]. A total of 10,584 participants with T2DM and CKD (eGFR 25–60 mL/min/1.73 m^2^) were randomized to sotagliflozin or placebo. Approximately 60% of trial participants had a baseline UACR value >30 mg/g. After a median follow‐up of 16 months, sotagliflozin was associated with a lower hazard for composite CV endpoint versus placebo (HR 0.74 [95% CI: 0.63–0.88]) [142]. However, subgroup analyses suggested that the effects of sotagliflozin on the composite CV endpoint were more pronounced in trial participants with baseline UACR values ≥30 mg/g (HR 0.67 [95% CI: 0.55–0.80]) versus <30 mg/g (HR 1.05 [95% CI: 0.76–1.46]) [142].

#### Clinical experience with MRAs

Steroidal MRAs (spironolactone and eplerenone) and ns‐MRAs (finerenone) have shown a benefit in patients with heart failure. In an analysis of patients with HFpEF who participated in the TOPCAT clinical trial, spironolactone reduced the UACR by 39% at 1 year (vs. baseline UACR [placebo adjusted]), with a more pronounced UACR‐reducing effect observed in those with severely increased UACR at baseline (76% UACR reduction) (Table 1) [143]. Thus, this medication class may have greater effects with increased UACR. Testing for UACR may allow clinicians to select patients most likely to benefit from treatment based on their pretreatment UACR. Moreover, this analysis of TOPCAT data demonstrated that UACR lowering was associated with a reduction in several adverse cardiovascular events, including a reduction in hospitalization for HF, although this may be partly attributed to the drug's systolic blood pressure–lowering effect. Use of eplerenone also lowers both UACR and systolic blood pressure levels to a greater amount when compared with placebo. In an analysis of the MIRAD clinical trial, high‐dose eplerenone decreased the UACR by 34% versus placebo at week 26 (compared with baseline) in patients with T2DM and at high risk of, or with, CVD (Table 1), although the effect may be attributed to eplerenone's blood pressure–lowering capabilities [144]. Although the EMPHASIS‐HF trial investigated the effect of eplerenone on HF‐related outcomes in individuals with a reduced LVEF (composite of death from cardiovascular causes or hospitalization for HF) [145], no sub‐analysis based on UACR at baseline has been performed. Finerenone recently received an HF indication by the FDA (2025) to reduce the risk of cardiovascular death, hospitalization for HF, and urgent HF visits in adult patients with HF with an LVEF ≥40% [128] based on the results of the FINEARTS‐HF trial [146]. In the FINEARTS‐HF trial, patients with HFpEF or HF with mildly reduced ejection fraction (HFmrEF) (LVEF ≥40%) received finerenone or matching placebo plus usual therapy. Finerenone was associated with a significantly lower rate of the composite of total worsening HF events and death from cardiovascular causes versus placebo (primary outcome) [146]. In the subgroup analysis of the primary outcome based on baseline UACR, the effect was similar in the <30 versus the >30 mg/g groups, suggesting a consistent effect across UACR categories but with a trend to greater effect in the lower UACR group (Table 1) [146].

Assessing the UACR at baseline and after initiating therapy can help determine treatment response. If the UACR does not decrease after initiating therapy (target is at least 30% decrease from baseline), initiation of other interventions should be considered. However, other aspects need to be considered in addition to a response marker, such as side effects of the drugs, concomitant medicines, comorbidities, and patient preference.

## Conclusions

Research in areas, such as proteomics, metabolomics, and transcriptomics, has attempted to identify new biomarkers that have the potential to be used in the analysis of kidney function and kidney disease progression [147, 148]. However, assessment of albuminuria, typically via the UACR (along with assessment of the glomerular filtration rate), remains the cornerstone of diagnosis and risk stratification of CKD in daily clinical practice [18]. Furthermore, studies and various analyses suggest that the UACR is a reliable biomarker for future cardiovascular and kidney risk as well as drug treatment response in different populations, including those with and without CKD. Using the UACR to help guide treatment means that this biomarker may be used to personalize medicine when used in conjunction with other patient factors such as presence of comorbid diabetes. Owing to the UACR being a reliable predictor of future diabetic kidney disease and cardiovascular events, urinary albumin levels should be checked in all at‐risk patients, such as those with diabetes and/or hypertension, at least once per year, as well as for those starting a new drug treatment for CKD or continuing treatment. Patients with high UACR have a high risk for both kidney and CV events, even with the use of new therapies [149], and need close monitoring. Many of the drugs that have shown efficacy in CKD and/or heart failure appear to exert their maximal benefit at higher baseline UACRs (typically patients already taking/or have taken maximally tolerated RAAS blocker) or at least exert positive effects across all UACR categories (usually from moderately increased UACR values). Finally, tests other than the UACR are available for measuring urinary albumin, but the UACR spot test is probably the most sensitive and specific test and is most convenient for patients, and so should be considered for use in clinical practice for measuring urinary albumin levels.

## Conflict of interest statement

HK has acted as an educational consultant for Bayer and is a member of a Data Safety and Monitoring Board for Alexion Pharmaceuticals. GLB was supported by T32 NIH grant DK07011 and was a consultant and/or steering committee member for clinical trials to Bayer, Janssen, KBP Biosciences, Ionis, Alnylam, AstraZeneca, Novo Nordisk, E‐STAR, and InREGEN. GLB was also editor of *Am J Nephrology*.

## Funding information

Medical writing support was provided by Lisa Moore, PhD, of Envision Catalyst of Envision Medical Communications, part of Envision Pharma Group, and this support was funded by Bayer Corporation.

## Supporting information




**Supporting Information**: Plain Language Summary (text) and (Figure), authors’ perspectives regarding guideline‐directed therapy in CKD associated with T2DM. CKD, chronic kidney disease; CV, cardiovascular; eGFR, estimated GFR, glomerular filtration rate; GLP‐1 RA, glucagon‐like peptide‐1 receptor agonist; ns‐MRA, nonsteroidal mineralocorticoid receptor antagonist; RAAS, renin–angiotensin–aldosterone system; SGLT2, sodium‐glucose cotransporter‐2; UACR, urinary albumin‐to‐creatinine ratio.

## Data Availability

Data sharing is not applicable to this article as no datasets were generated or analyzed during the current study.
