# Supplementary material for: The urinary albumin‐to‐creatinine ratio can direct personalized prevention and treatment for cardiovascular and chronic kidney disease
Source: J Intern Med. 2026 Feb 3;299(4):444–66. doi: 10.1111/joim.70066 (PMC12950637; doi:10.1111/joim.70066)

**Supporting Information**

**Plain Language Summary (text)**

People with damaged kidneys may have proteins in their urine (“pee”) that are normally only found in the blood. These proteins end up in urine because damaged kidneys will eventually become “leaky.” Protein in urine can be a complication of having diabetes or high blood pressure. Albumin is one of these proteins. Creatinine is the name of a chemical produced from muscle movement that is found as a waste product in urine. A type of test that divides how much albumin is in urine by how much creatinine is in urine (producing a ratio called the urinary albumin-to-creatinine ratio [UACR for short]) can be used to help decide how badly damaged the kidneys are. A person with a UACR of 30 up to 300 milligrams per gram has a moderately increased amount of urine albumin, and a person with a UACR of more than 300 milligrams per gram has a severely increased amount of urine albumin. Having a moderately or severely increased UACR increases a person’s risk of developing heart disease and worsening of existing kidney problems. Drug treatments are often personalized, which means that drugs are chosen based on a person’s health circumstances, and drug treatments can be personalized based on the UACR. A reduction in the UACR by at least 30% after starting drug treatment indicates lower risk for kidney disease progression and a reduction in the risk of developing heart and circulation problems.

**Methods for literature review**

PubMed searches were used to find applicable articles for inclusion in the review. The following search terms were used in three separate searches: (“personalized OR personalised”) AND (“medicine OR care OR healthcare”) AND (“kidney OR renal”); (“UACR OR albumin* OR creatinine”) AND (“heart OR cardio*”); (“UACR OR albumin* OR creatinine”) AND (“renal OR kidney”) AND (“biomarker OR marker OR predict*”). Searches were limited to title only, English, humans, and past 5 years (1 January 2018–31 December 2023). These initial searches produced 375 articles. The search results were downloaded from PubMed into the EndNote 9.3.3 program, where the articles were screened using EndNote’s search features as follows: removal of duplicate articles; review of article titles and removal of nonrelevant articles; review of full text and removal of nonrelevant articles. Nonrelevant articles removed included the following types of article: cancer or carcinoma; child, pediatric, and neonate; genetic bench studies; and serum albumin focus. Completed phase III, phase IV, and observational studies listed in ClinicalTrials.gov in applicable fields were also reviewed, and associated primary or secondary manuscripts were accessed and included in the reference list and cited where appropriate. Additional papers not included in the original PubMed searches were also included if recommended by the authors. Clinical treatment guideline articles applicable to CKD, diabetes, and/or hypertension were also included with the reference list.

**Supplementary Figure.** Authors’ perspectives regarding guideline-directed therapy in CKD associated with T2DM. [For baseline <30 mg/g, SGLT2 inhibitor is included under “Treat” because there is no stated baseline UACR value for SGLT2 inhibitor treatment]. CKD, chronic kidney disease; CV, cardiovascular; eGFR, estimated GFR, glomerular filtration rate; GLP-1 RA, glucagon-like peptide-1 receptor agonist; ns-MRA, nonsteroidal mineralocorticoid receptor antagonist; RAAS, renin‒angiotensin‒aldosterone system; SGLT2, sodium-glucose cotransporter-2; UACR, urinary albumin-to-creatinine ratio.


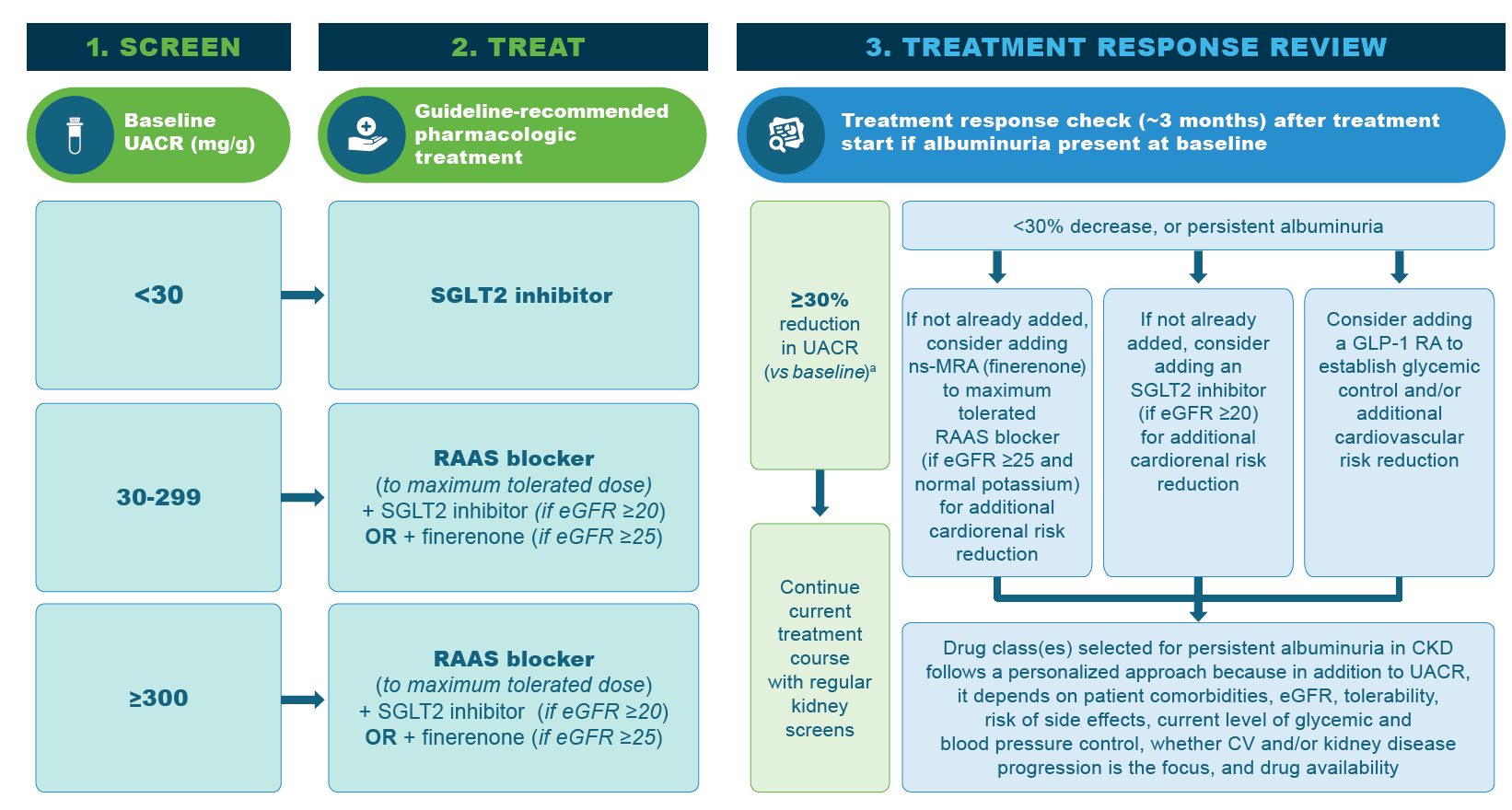

Supplement: Supplementary file 1 — Supporting Information: Plain Language Summary (text) and (Figure), authors’ perspectives regarding guideline‐directed therapy in CKD associated with T2DM. CKD, chronic kidney disease; CV, cardiovascular; eGFR, estimated GFR, glomerular filtration rate; GLP‐1 RA, glucagon‐like peptide‐1 receptor agonist; ns‐MRA, nonsteroidal mineralocorticoid receptor antagonist; RAAS, renin–angiotensin–aldosterone system; SGLT2, sodium‐glucose cotransporter‐2; UACR, urinary albumin‐to‐creatinine ratio. [file JOIM-299-444-s001.docx]
